# Physiological, Biochemical and Gene Expression Analyses of *Halimodendron halodendron* Responding to Drought Stress

**DOI:** 10.3390/genes16111274

**Published:** 2025-10-28

**Authors:** Huanqiong Hu, Panpan Zhang, Ling Wang, Hailian Liang, Jiye Liang, Ruiheng Lyu

**Affiliations:** 1 College of Horticulture and Forestry, Tarim University, Alar 843300, China; hhq530536195@163.com (H.H.); zpp1011@taru.edu.cn (P.Z.); wl18215118649@163.com (L.W.); hailian18277545041@163.com (H.L.); 2 School of Pharmacy, Youjiang Medical University for Nationalities, Baise 533000, China

**Keywords:** drought resistance characteristics, Illumina sequencing, genes, photosynthesis

## Abstract

**Background:** As a typical xerophyte, *H. halodendron* can not only grow in desert sandy areas but also serves as an excellent nectar source and ornamental plant. However, research on its molecular and physiological mechanisms underlying drought tolerance remains limited. **Methods**: This study systematically investigated its drought resistance characteristics by integrating physiological parameters and Illumina transcriptome sequencing, and further validated key genes involved in the drought resistance mechanisms. **Results**: A total of 46,305 functional genes were identified, among which 6561 were differentially expressed genes (DEGs). These DEGs were significantly enriched in chloroplast function, photosynthesis, proline biosynthesis, and peroxidase activity. Under drought stress, the net photosynthetic rate, stomatal conductance, chlorophyll content, and transpiration rate decreased. Under severe drought conditions, only 5 out of 80 photosynthesis-related DEGs were up-regulated, while the rest were down-regulated, indicating that reduced chlorophyll content impaired light absorption, carbon reactions, and photosynthetic efficiency. Additionally, the contents of proline, soluble sugars, and soluble proteins, as well as the activities of superoxide dismutase (SOD), catalase (CAT), and peroxidase (POD), increased. The identification of 35 osmotic regulation-related and 39 antioxidant enzyme-related DEGs suggests that *H. halodendron* enhances osmotic adjustment substance synthesis and reactive oxygen species (ROS) scavenging capacity to counteract osmotic stress. **Conclusions**: Physiological, biochemical and gene expression analyses under drought stress provide a basis for the study of the drought tolerance characteristics of *H. halodendron*, which is of great significance for ecological environment governance using *H. halodendron.*

## 1. Introduction

Drought stress is a major constraint on crop productivity, and its impact exceeds the combination of all other factors affecting crop production [1]. The Intergovernmental Panel on Climate Change (IPCC) reported that the average temperature will rise by 1.8–4.0 °C in 2100, and most regions of the world will face drought, which will pose a great threat to food production and economic development [1]. The Tarim Basin is located in the hinterland of the Eurasian continent, with its edge being the gravel Gobi connected to mountains, and its center being a vast desert. It has severe sandstorms all year round and little precipitation. Coupled with human factors such as unreasonable fertilization, irrigation and industrial pollution, the groundwater level continues to decline, the greening status continues to deteriorate, and vegetation continues to decrease. The Tarim Basin in China is currently facing the most severe drought and has the most fragile ecological environment among desert ecosystems [2]. It is crucial to address the issues stemming from soil drought in this region.

Drought stress is one of the most severe environmental factors affecting plant growth, survival and regeneration [3]. Lack of water in cells can cause changes in plant morphology, decrease in photosynthetic activity, increase in reactive oxygen content, and metabolic disorders [4]. In order to reduce the impact of drought on plant growth and avoid cell dehydration, plants have evolved a variety of means to deal with drought stress. These include increasing water absorption through developed roots and synthetic osmotic regulating substances; increasing water conduction by increasing vascular cross-sections and leaf veins; limiting transpiration by closing stomata; increasing cuticle thickness; changing leaf shape, size, and number; improving water storage by increasing the fleshiness of leaves and stems; and eliminating reactive oxygen species free radicals by increasing antioxidants and maintaining and stabilizing cell membranes. Additionally, all of these methods are genetically regulated. Therefore, studying the physiological, biochemical and molecular regulation of plants under drought stress is of great significance to cope with the threat posed by drought.

*H. halodendron* is a perennial shrub of the genus *Halimodronin,* belonging to the leguminous family. It has strong drought tolerance and grows well in deserts. Studies have shown that the ability of *H. halodendron* seeds to germinate is not completely inhibited under severe drought [5]. The plant’s leaves have a high water absorption rate and water accumulation, which helps to repair xylem plugs, improve water conduction efficiency, and enhance drought tolerance [6]. In addition, *H. halodendron* contains high protein and is a favorite forage for desert livestock such as camels and sheep. Its flowers have extremely high ornamental value and can be used as hedges. They are also a good honey source and have high ecological value, economic value and ornamental value [7]. It has good adaptability in ecologically fragile environments [8], so it is an excellent tree species used to prevent wind and sand fixation and improve the environment, and has positive significance for ecological restoration.

However, there are few studies on the drought-resistant physiological characteristics, organizational structure and molecules of *H. halodendron*. Therefore, in this study, aiming to contribute to the current vegetation restoration and protection efforts in sandy areas, we selected *H. halodendron* from the desert frontier of the Tarim Basin and conducted physiological, biochemical and molecular research on its drought tolerance to understand its drought adaptation mechanism. We aim to provide a reference for the selection of drought-resistant species in the future ecological environment management process in desert areas and a reasonable basis for later seedling cultivation, afforestation, and water allocation.

## 2. Materials and Methods

### 2.1. Cultivation and Treatment of Plant Materials

The seeds of *H. halodendron* used in this study originated from the northern fringe of the Taklimakan Desert (81°27′ E, 40°54′ N), a region characterized by a warm temperate continental arid desert climate. In February, plump and pest-free seeds were selected, soaked in warm water at 60 °C for 24 h, and sown in the greenhouse of the Horticultural Experimental Station at Tarim University. After three months of germination and growth, uniform seedlings with comparable plant height and ground diameter were selected and transplanted into pots. The growth substrate was prepared by mixing washed river sand, garden soil, and potting substrate in a 3:1:1 ratio. Each pot contained 5 kg of dry soil, with a total nitrogen content of 3.33 g·kg^−1^, organic matter content of 55.58 g·kg^−1^, available potassium content of 0.18 g·kg^−1^, and available phosphorus content of 0.47 g·kg^−1^. After transplanting, the seedlings were watered adequately and placed outdoors under full sunlight. To promote robust growth, they were irrigated every 15 days with a half-strength Hoagland nutrient solution.

Water stress treatments were established based on the field soil moisture content (20.31%) as follows (Table 1):

CK (Control): 80–90% soil moisture;

W_1_ (Mild drought): 60–70% soil moisture;

W_2_ (Moderate drought): 40–50% soil moisture;

W_3_ (Severe drought): 20–30% soil moisture;

Each treatment consisted of 4 groups, with 3 pots per group, resulting in 12 pots per treatment and 48 pots in total. Two days prior to the initiation of the treatments, all potted plants were fully irrigated. Soil moisture content was monitored daily until the target levels were reached. Thereafter, moisture was maintained by daily weighing and replenishing water between 19:00 and 20:00.

After 30 days of treatment, mature leaves without pests and diseases (the 3rd to 5th leaf from the top end of the morphology) were selected and sampled to determine physiological and biochemical indicators. Some leaves were then selected from the mature leaves and placed in a refrigerator at −80 °C for storage.

### 2.2. Determination of Growth Parameters

At the onset of stress (day 0), the initial ground diameter (measured with a 0.1 mm precision vernier caliper), plant height, and number of new branches were recorded for all plants. Three new branches per plant were tagged, and their initial lengths were measured using a ruler with 0.1 mm accuracy. After 30 days of stress treatment, the same parameters—ground diameter, plant height, new branch length, and number of new branches—were measured again to calculate the increments.

On day 0, three plants were randomly selected for destructive sampling. Their roots, stems, and leaves were separated, rinsed with deionized water, and dried in an oven at 80 °C for 72 h. The dry mass of each tissue was then determined using a balance with 0.001 g accuracy. After 30 days of stress, another three plants were randomly sampled, and the same procedure—separation, washing, drying, and weighing—was repeated to determine the final dry weights.

At day 0 of stress, the initial ground diameter (precision 0.1 mm vernier caliper), initial plant height and initial number of new branches of all plants were measured. We selected 3 new branches from each plant to be labeled and measured the initial new branch length (accuracy: 0.1 mm ruler). After 30 days of stress, ground diameter, plant height, new branch length and number of new branches were measured again to calculate the increase.

At day 0, 3 plants were randomly selected to separate the roots, stems, and leaves. They were washed with deionized water, dried in an oven at 80 °C for 72 h, and weighed on a balance (accuracy 0.001). After 30 days of stress, another 3 plants were randomly selected, and the roots, stems, and leaves were separated, washed, dried, and weighed again. The dry weight was then calculated.

### 2.3. Photosynthetic Parameter Measurement

We selected 3–6 mature healthy leaves on the upper end of the plant to measure photosynthetic parameters using red and blue light sources of an LI-COR portable photosynthesis instrument (LI-6400XT, LI-COR Biosciences, Lincoln, NE, USA). The light intensity was set to 1500 μmol·m^−2^·s^−1^, the temperature was set to 35 °C, and the air flow rate was set to 500 μmol·s^−1^. A buffer bottle was used to control the CO_2_ concentration in the reference room to be consistent with the external environment. The net photosynthetic rate (Pn), transpiration rate (Tr), and stomatal conductance (Gs) were measured on sunny days from 10:00 to 11:00 [9]. After completing the measurement, we harvested the leaves to measure the leaf area and calculated the photosynthetic parameters to determine the water use efficiency (WUE) as Pn/Tr.

### 2.4. Chlorophyll Content Determination

Chlorophyll a (Chl a), chlorophyll b (Chl b), and total chlorophyll content were calculated using a spectrophotometer. To do this, 0.1 g of fresh leaves was taken, ground in an ice bath, and transferred to a test tube, and 10 mL of 95% ethanol was added. The absorbance at 663 and 645 was then measured using a spectrophotometer.

### 2.5. Determination of Relative Moisture Content and Electrical Conductivity

We randomly selected fresh leaves, weighed them on an electronic balance, and recorded the fresh weight W_1_. Then we completely immersed the leaves in distilled water until saturated and weighed them to obtain a fresh weight of W_2_. Finally, the leaves were placed in an oven at 105 °C for de-activation for 30 min, and then dried at 60 °C to constant weight to obtain dry weight W_3_. The calculation formula for leaf relative water content (RWC) was:

(1) $$RWC=\frac{\left(W_{1}-W_{3}\right)}{\left(W_{2}-W_{3}\right)}100\%$$

The conductivity (REC) was calculated using a conductivity meter (DDS-11AGA, TBTSCIETECH, Nanjing, China). We weighed 0.1 g of chopped leaves (excluding main veins), soaked them in 10 mL of distilled water for 12 h, and then measured the electrical conductivity R1 of the liquid. After boiling for 20 min and cooling to room temperature, the conductivity R2 was measured and REC (%) = R1/R2 × 100% was calculated.

### 2.6. Determination of Antioxidant Enzyme Activity and Superoxide Anion (O_2^−^_) Production Rate

0.1 g of fresh leaves was taken, rinsed with distilled water, and dried with filter paper. The leaves were then placed into 2 mL Eppendorf tubes and stored in an ultra-low temperature refrigerator for the analysis of superoxide dismutase (SOD) activity, peroxidase (POD) activity, and catalase (CAT) activity [10]. These activities were measured using a kit (Suzhou Keming, micromethod, Suzhou, China), with absorbance values recorded at OD560, OD470, and OD240. The rate of O_2^−^_ production was determined using the superoxide anion kit (Suzhou Keming, Oxygen free radical). We referred to the instruction manual of the kit for the specific steps.

### 2.7. Determination of Soluble Substance

0.1 g of fresh leaves was taken, rinsed with distilled water, and then dried with filter paper. The leaves were placed in 2 mL Eppendorf tubes and stored in an ultra-low temperature refrigerator for the detection of proline (Pro) content, soluble sugar (SS) content, and soluble protein (SP) activity [11]. All the tests were performed using the kit (Suzhou Keming, micro method), and we referred to the instruction manual of the kit for the specific steps.

### 2.8. Microstructure

In order to facilitate the observation of the microscopic structure, the paraffin sectioning method employed by Yao et al. [12] was adopted for sample preparation. Leaves with a maturity of 5 mm in length and 4 mm in width were cut and placed in FAA fixative for over 24 h. Subsequently, the leaves were transferred to various ethanol gradients for dehydration, followed by immersion in a mixture of xylene and ethanol to achieve transparency. The transparent leaves were placed in melted paraffin and kept warm in a wax melting box. Embedding was performed after the paraffin wax was completely immersed in the tissue. The embedded wax blocks were fixed on a Microtom (RWD Life Science, Shenzhen, China) to make thin slices with a thickness of 5–8 μm. The slices were attached to the slides and dried in a 45 °C thermostat (CTHI-150(A)B, Shanghai Shidukai Instrument & Equipment Co., Ltd., Shanghai, China). The slides were dewaxed by applying xylene, after which they were stained with 1% Senna for more than 4 h. After staining, the sections were dehydrated with pure alcohol and then made transparent by xylene. Transparent sections were coated with drops of Canada gum and sealed with coverslips. Photographs were taken under a digital microscope (OPLENIC), and the corresponding data were recorded (all the above chemical reagents were purchased from Dingyuan Biotechnology Co., Ltd., Guangzhou, China).

### 2.9. Ultrastructure

Fresh leaves measuring 1 × 1 mm were first rinsed with 0.1 mol·L^−1^ PBS buffer and then fixed in 2.5% glutaraldehyde overnight. They were subsequently rinsed three times with 0.1 mol·L^−1^ PBS buffer for 15 min each time. The leaves were then fixed with osmic acid for 2–4 h and dehydrated through a series of solutions including 30%, 50%, 70%, 80%, 90%, and 100% acetone, followed by acetone: ethanol (1:1), pure acetone, and resin: acetone (1:3). After gradient dehydration with acetone (1:1), the ultrathin sections were prepared by embedding in resin overnight and stained with 2% uranium acetate for 40 min and 3% lead citrate for 3 min. Finally, the sections were observed using transmission electron microscopy (JEM-2100Plus) [13]. (All the above chemical reagents were purchased from Dingyuan Biotechnology Co., Ltd.)

### 2.10. Extraction and Identification of Total RNA

The CK and W3 treated samples underwent transcriptome sequencing. Total RNA was extracted from H. halodendron using Trizol reagent (DiNing, Beijing, China). RNA degradation and contamination were monitored by UV spectrophotometer and 1% agarose gel electrophoresis. RNA concentration was measured using the Qubit^®^ RNA Analysis Kit in Qubit^®^ 2.0 Fluorometer (LifeTechnologies, Carlsbad, CA, USA). RNA integrity was assessed using the RNANano2100 assay kit in the Bioanalyzer 6000 system (AgilentTechnologies, Santa Clara, CA, USA).

### 2.11. Illumina Library Preparation and Sequencing

1 μg of RNA per sample was taken as the input material for RNA preparation. Sequencing libraries were generated using the TruSeq RNA Library Kit (Illumina, San Diego, CA, USA), and index codes were added to each sample’s attribute sequence. The mRNA was purified from total RNA using poly-T oligo ligated magnetic beads. First-strand cDNA was synthesized with random hexamer primers and M-MuLV reverse transcriptase (RNaseH-). Second-strand cDNA synthesis was then carried out with DNA polymerase I and RNase H, and the remaining overhangs were converted to blunt-end by ribonucleic acid exonuclease/polymerase activity. DNA fragments 3′ end was adenylated, ligated to IlluminaAdaptor, and hybridized. The library fragments were purified with the AMPureXP (v3.20.5502) system (Beckman Coulter, Beverly, MA, USA) and screened for fragments between 150–200 bp in length. PCR was performed with Phusion High-Fidelity DNA polymerase, Universal PCR primers and Index (X) primers. Finally, the amplification products were purified (AMPureXP system), and the library quality was evaluated on an Agilent Bioanalyzer2100 system.

### 2.12. Stitching and Assembly of Illumina Sequencing Data

The original data (raw reads) obtained by sequencing were processed by FastQC. Reads with low quality (Q20 ≤ 80%), joint contamination, and high unknown base N content (Ns ≥ 5%) were filtered out. The clean reads were obtained by de novo assembly with Trinity.

### 2.13. Gene Annotation

Predicted high-quality protein information was annotated in six databases: GO (GeneOntology), eggNOG (cut-off—Evalue ≤ 1^e−3^), KEGG (Kyoto Encyclopedia of Genes and Genomes, cut-off—Evalue ≤ 1^e−5^), NCBI Non-Redundant Proteins (NR, cut-off—Evalue ≤ 1^e−5^), SwissProt (cut-off—Evalue ≤ 1^e−5^) and Pfam. NR, eggNOG, KEGG and SwissProt annotations of transcripts were obtained using Blastx and Diamond software (2.14.0 and 2.1.9). Based on the NR annotation results, GO annotation and classification were performed in the Blast2GO (6.0 Mac platform) program. The annotated genes were classified with the eggNOG function, enriched with KEGG, and analyzed with GO.

### 2.14. Quantification of the Gene Expression Levels, Identification, and Function Analysis of DEGs

The gene expression level of each sample was determined using RSEM. The clean data from Illumina sequencing was mapped to SMRT sequencing data, and the read count for each gene was obtained based on the mapping results. Differentially expressed genes are those with an average count greater than 5, a selection difference of more than 2 times (|log2FC| Genes with ≥1), and FDR < 0.05 based on gene data.

Differential expression analysis is performed using DeSeq2 to determine differential expression between drought treatment and CK. For function annotations, GO enrichment analysis is performed on DEGs using the GOseqR package (Bioconductor,1.2.1). KEGG enrichment analysis of DEGs was performed using KOBAS software (Bioconductor,1.2.1).

### 2.15. Data Processing

The test data were statistically processed using Microsoft Excel 2016 software, SPSS26.0 software was used for multiple comparisons (α = 0.05), and Origin2022 software was used for plotting. The data in this paper are expressed as mean ± standard deviation.

## 3. Result

### 3.1. Effects of Drought Stress on Growth of H. halodendron

Under drought stress, the growth of *H. halodendron* was affected to different degrees, and the growth became slower with the prolongation of stress time (Table 2). As the drought degree increased, the growth of plant height, ground diameter, number of new branches and length of new branches of *H. halodendron* gradually decreased. Under mild, moderate, and severe drought stress, the plant height increment and new branch length increment of *H. halodendron* were significantly reduced compared to the control group (CK). The plant height increment decreased by 37.50%, 58.45%, and 75.00%, respectively, while the new branch length increment decreased by 19.30%, 32.89%, and 70.18%, respectively. However, there was no significant difference in the number of new branches. The ground diameter increment under severe drought treatment showed a significant difference from the control group, with a decrease of 76.28%.

As shown in Table 3, at 30d of drought stress, the dry weight of the root gradually decreased with the increase of the stress level. The root dry weight decreased by 39.09% under severe drought stress, which was significantly different from that of CK; however, the stem dry weight, leaf dry weight, and whole plant dry weight increased by 20.11%, 7.40%, and 3.37%, respectively, compared with that of CK under mild drought stress. These values were all significantly lower than that of CK, which decreased by 42.77%, 49.13%, and 44.20%, respectively, under severe drought stress.

### 3.2. Effects of Drought Stress on Photosynthetic and Physiological Characteristics of H. halodendron

**Figure 1:**
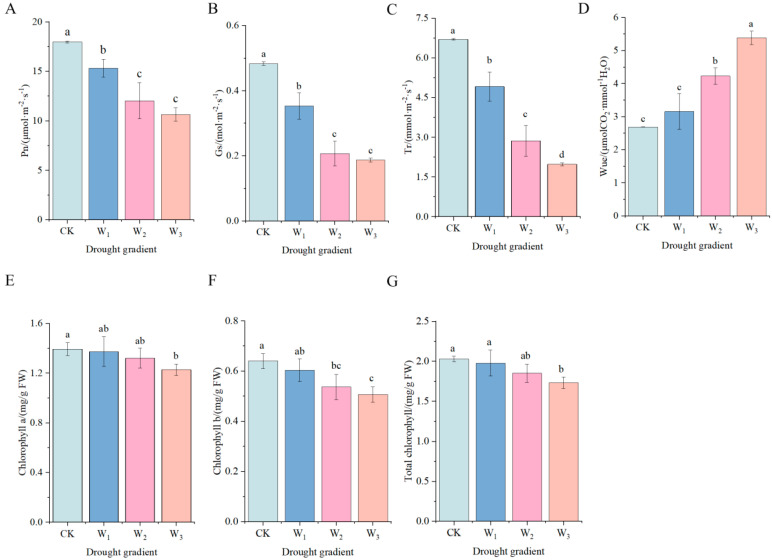
Effects of drought stress on photosynthetic characteristics of *H. halodendron*. (**A**) photosynthetic rate. (**B**) stomatal conductance. (**C**) transpiration rate. (**D**) water use efficiency. (**E**) chl a content. (**F**) chl b content. (**G**) total chlorophyll content. Note: Analysis of variance (ANOVA) was used as the statistical method. Different lowercase letters indicate significant differences between treatments (*p* < 0.05).

Figure 1A–C show that the net photosynthetic rate, stomatal conductance, and transpiration rate of *H. halodendron* decreased significantly compared to the control (CK) after 30 days of drought stress (*p* < 0.05). Specifically, the net photosynthetic rate decreased by 14.79%, 33.10%, and 40.82% under mild, moderate, and severe stress levels, respectively, with the lowest rate recorded at 10.63 μmol·m^−2^·s^−1^ under severe stress. Similarly, stomatal conductance decreased by 26.90%, 57.23%, and 61.37% under mild, moderate, and severe stresses, respectively, with the lowest value at 0.19% under severe stress.mol·m^−2^·s^−1^. Transpiration rate was reduced by 26.63%, 57.24%, and 70.48%, respectively, compared with CK, and was the lowest at 1.98 mmol·m^−2^·s^−1^ under severe stress. Water use efficiency was significantly higher and was the highest under severe stress (5.39 μmol CO_2_·mmol^−1^ H_2_O), elevated compared with CK by 100.75% (Figure 1D).

### 3.3. Effects of Drought Stress on Chlorophyll Content of H. halodendron

As shown in Figure 1E–G, the changing pattern of photosynthetic pigment content of *H. halodendron* leaves with drought gradient is depicted. At 30 days of drought stress, the chla, chlb, and total chlorophyll contents showed a steady state under mild and moderate treatments, with no significant difference compared with the control group (CK), and were significantly lower than CK under severe drought stress.

### 3.4. Effects of Drought Stress on Drought-Resistant Physiological Characteristics of H. halodendron

**Figure 2:**
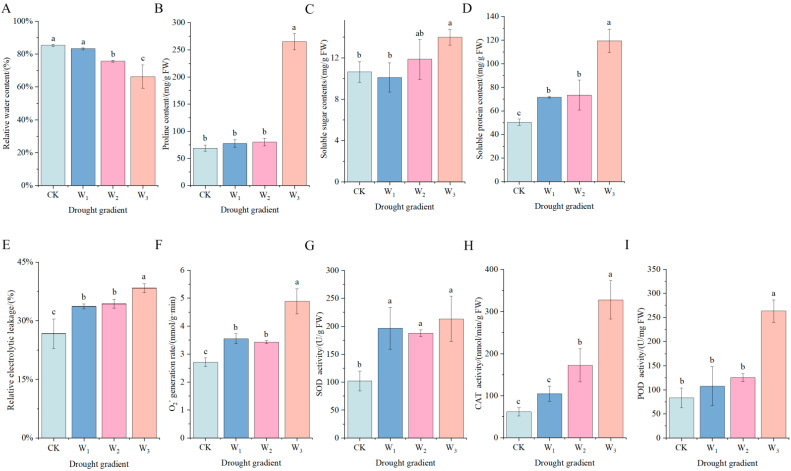
Effects of drought stress on osmotic regulation and antioxidant properties of *H. halodendron*. (**A**) Relative water content. (**B**) proline content. (**C**) soluble sugar content. (**D**) soluble protein content. (**E**) Relative conductivity. (**F**) O_2^−^_ production rate. (**G**) SOD activity. (**H**) CAT activity. (**I**) POD activity. Note: Analysis of variance (ANOVA) was used as the statistical method. Different lowercase letters indicate significant differences between treatments (*p* < 0.05).

As shown in Figure 2A, at 30 days of drought stress, the relative water content of *H. halodendron* was significantly lower (*p* < 0.05) than that of the control at moderate and severe stresses. Specifically, it was 11.33% lower under moderate stress and 22.27% lower under severe stress compared to the control.

As shown in Figure 2B–D, at 30d of drought stress, the Pro content was not significantly different from CK under mild and moderate stress, and was significantly higher under severe stress, with an increase ratio of 282.85%. The soluble sugar content of *H. halodendron* increased by 31.57% under severe drought stress compared to CK. Soluble protein content gradually increased with the increase of drought stress, and all three stress treatments were significantly different from CK, with 41.93%, 45.71% and 137.00%, respectively.

### 3.5. Effects of Drought Stress on Antioxidant Properties of H. halodendron

As shown in Figure 2E, the relative electrical conductivity of *H. halodendron* significantly increased under the three stresses, and was 26.25%, 28.75% and 43.75% higher than that of CK, respectively. As shown in Figure 2F, O_2^−^_ production rate also gradually increased under the three drought stresses. Compared with CK, it increased by 30.84%, 26.29% and 80.22%, respectively.

The SOD, POD and CAT activities of *H. halodendron* played important roles in antioxidant defense under drought stress (Figure 2G–I). The SOD, POD and CAT activities of *H. halodendron* showed an increasing trend with the increase of drought stress for 30d. The SOD activity was significantly higher than that of CK under drought stress, with an increase of 92.12%, 83.40% and 108.50% under the three kinds of drought stresses, respectively. CAT activity was significantly higher than CK under moderate and severe drought stress, increased by 178.89% and 430.67%, respectively.

### 3.6. Effects of Drought Stress on Leaf Structure of H. halodendron

**Figure 3:**
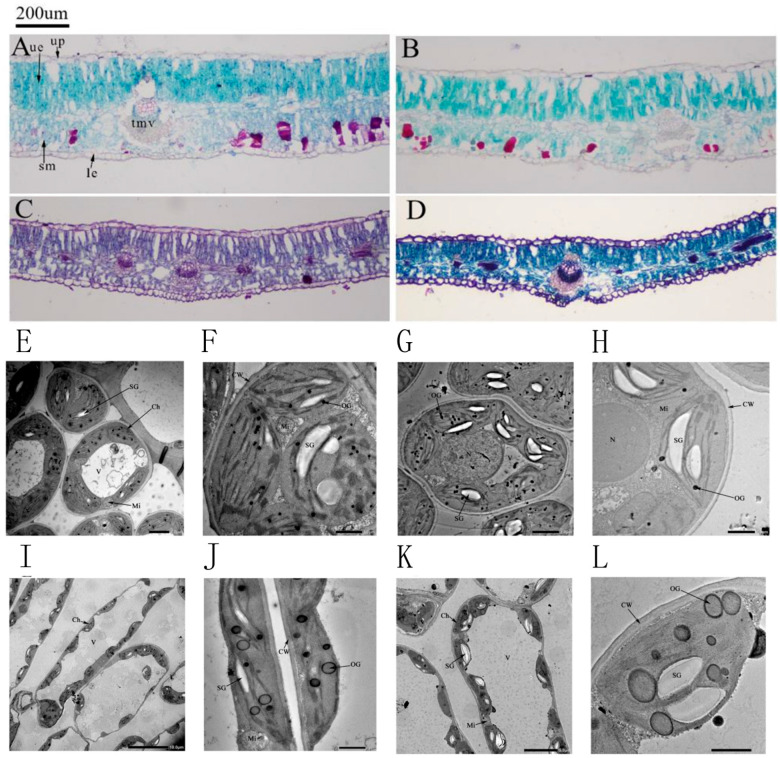
Effects of drought stress on the leaf structure of *H. halodendron* are clearly depicted. (**A**) Leaf microstructure of control group (CK). (**B**) Leaf microstructure of mild drought group (W1). (**C**) Leaf microstructure of moderate drought group (W2). (**D**) Leaf microstructure of severe drought group (W3). (**E**,**F**) Leaf ultrastructure of CK. (**G**,**H**) Leaf ultrastructure of W1. (**I**,**J**) Leaf ultrastructure of W2. (**K**,**L**) Leaf ultrastructure of W3. Note: (**A**): CK (30d); (**B**): W1 (30d); (**C**): W2 (30d); (**D**): W3 (30d) (**E**): CK × 600 (30d); (**F**): CK × 2000 (30d); (**G**): W1 × 800 (30d); (**H**): W1 × 3000 (30d); (**I**): W2 × 600 (30d); (**J**): W2 × 2500 (30d); (**K**): W3 × 1500 (30d); (**L**): W3 × 3000 (30d). Ue: upper epidermis; up: palisade tissue; le: lower epidermis; sb: vesicle structure; cr: Crystal cell; kmc: Garland cell; tmv: midvein thickness; Ch: chloroplast, V: vacuole, Mi: mitochondria, N: nucleus, Nu: nucleolus, OG: osmiophilic granules, SG: starch granules, GL: grana lamella, SL: stroma lamella, CW: cell wall.

Figure 3 shows the microscopic structure of the leaf under different environments. The micrograph reveals that the cellular architecture of *H. halodendron* leaf comprises the epidermis, lower epidermis, spongy tissue, palisade tissue, vascular bundles, and stomata. The palisade tissue consists of 2–3 layers of closely packed parenchyma cells, whereas the spongy tissue exhibits an irregular morphology, formed by 3–4 layers of loosely arranged cells.

Figure 3B–D present the leaf microscopic structures after 30 days of mild, moderate, and severe drought stress, respectively. Combined with the data in Table 4, compared with the Control (CK), both leaf thickness and palisade tissue thickness increased significantly under mild drought stress by 7.58% and 4.49%, respectively. In contrast, these parameters decreased markedly under moderate and severe stress. The thickness of the spongy tissue declined significantly with increasing drought intensity, showing reductions of 23.65%, 51.65%, and 67.13% compared to the CK group. Similarly, the thickness of the main vascular bundle exhibited a comparable trend to leaf and palisade tissue thickness. It increased substantially under mild drought stress, peaking at a value 43.51% higher than that of the CK group, and then progressively decreased with increasing drought severity, with the differences being statistically significant. Furthermore, the staining intensity of the microtissues deepened as the drought stress intensified. The darkest and most intense red coloration was predominantly localized within the vascular bundle regions. As the stress progressed, the vascular bundles became thicker and exhibited more intense red staining, indicating a significant enhancement in the lignification of the xylem vessels and surrounding fiber cells. This adaptation likely serves to ensure the integrity and prevent the collapse of the water transport conduits under extreme water deficit.

As shown in Figure 3, drought stress at 30 days had varying effects on the cell structure and chloroplast structure of the leaf blades of *H. halodendron*. Figure 3E,F show the structure of the chloroplasts and organelles in the control group of *H. halodendron*. The cells appeared as smooth ovals, the chloroplasts were distributed in a spindle shape attached to the cell wall, and the mitochondria were distributed around the chloroplasts. The basal lamellae of chloroplasts were closely and neatly arranged, with a few osmiophilic granules and starch granules scattered inside. In Figure 3G,H, it is shown that under mild drought stress, the chloroplasts of *H. halodendron* were irregularly shaped and distributed far from the cell wall. Additionally, the size and volume of starch granules inside the chloroplasts increased, along with an increase in the number of osmiophilic granules. The basal lamellae of the vesicle-like body were elongated and partly loose. Figure 3I,J demonstrated that under moderate drought stress, the cell wall of *H. halodendron* was bent, the chloroplasts were twisted and deformed, positioned far from the cell wall, partially free from the wall, and some of the mitochondria were distributed around the chloroplasts. Additionally, it was observed that the basal lamellae of the cyst-like bodies were loosely stacked, the osmiophilic granules increased in size and number, and became lighter in color. Figure 3K,L shows that under severe drought stress, the chloroplasts of the thorns of *H. halodendron* have a variety of shapes. The chloroplasts contain more amylose granules and osmiophilic granules that increase in number, size, and gradually become hyaline.

### 3.7. Identification of Transcripts

To investigate gene transcription changes in *H. halodendron* under drought stress, next-generation sequencing (NGS) was employed to sequence leaf samples. After eliminating low-quality reads and reads containing adapters, a total of 31.34 GB of clean data were obtained. The clean data were de novo assembled into overlapping clusters, which were subsequently combined to yield a total of 152,729 high-quality transcripts. These transcripts exhibited a minimum length of 179 bp, a maximum length of 15,719 bp, an average length of 1017 bp, an N50 of 1888 bp, and a GC content of 40.29%. The Q30 value was 91%. Furthermore, from these transcripts, 46,305 functional genes were identified. These functional genes ranged in length from 201 bp to 15,719 bp, with an average length of 505 bp, an N50 of 1632 bp, and a GC content of 41.23%. The Q30 value for these functional genes was also 91% (Table 5).

### 3.8. Gene Annotation

**Figure 4:**
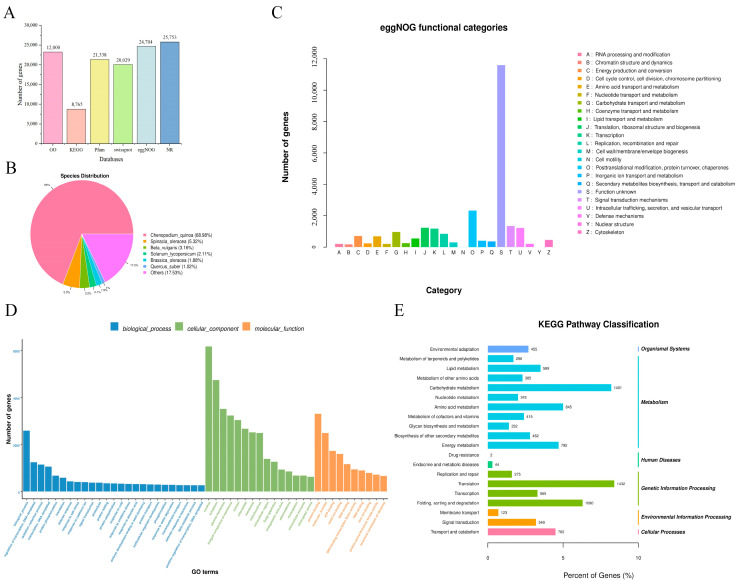
Annotation of the *H. halodendron* transcript. (**A**) The number of genes labeled in 6 databases is shown in the figure. (**B**) NR database annotation of the *H. halodendron* transcript. (**C**) eggNOG database annotation of the *H. halodendron* transcript. (**D**) GO database annotation of the *H. halodendron* transcript. (**E**) KEGG database annotation of the *H. halodendron* transcripts.

For a more comprehensive gene annotation, the 46,305 functional genes were annotated using GO, KEGG, NR, eggNOG, SwissProt, and Pfam databases. In SwissProt, 20,029 (43.25%) functional genes were annotated, while 21,338 (46.08%) functional genes were annotated in Pfam (Figure 4A). In the NR database, 25,753 (55.62%) functional genes were annotated, with 17,764 (68.98%) having various hits to quinoa. This was followed by hits to spinach (1370 bp, 5.31%), beet (813 bp, 3.16%), and tomato (543 bp, 2.11%) (Figure 4A,B).

Furthermore, eggNOG annotations showed that 24,704 (53.35%) functional genes were classified into 23 functional categories. The largest category was “posttranslational modification, protein turnover, chaperones” (2319 functional genes), followed by “signal transduction mechanisms” (1348 functional genes) and “translation, ribosomal structure and biogenesis” (1224 functional genes) (Figure 4A,C).

The GO database annotated a total of 23,220 (50.15%) functional genes (Figure 4A). These functional genes were primarily enriched in pathways related to “biological processes” “regulation of transcription, DNA-templated” “oxidation-reduction process” “transcription, DNA-templated” “nucleus” “cytoplasm” “plasma membrane” “cytosol” “chloroplast” “protein binding” “molecular function” “ATP binding” and “metal ion binding” (Figure 4D).

To explore the main biological processes in *H. halodendron*, 8765 (18.93%) functional genes were mapped to the KEGG database. Among these, the most enriched subcategories were “translation” (1432 functional genes); “carbohydrate metabolism” (1401 functional genes); and “folding, sorting and degradation” (1080 functional genes) (Figure 4A,E).

### 3.9. Differential Gene Analysis

**Figure 5:**
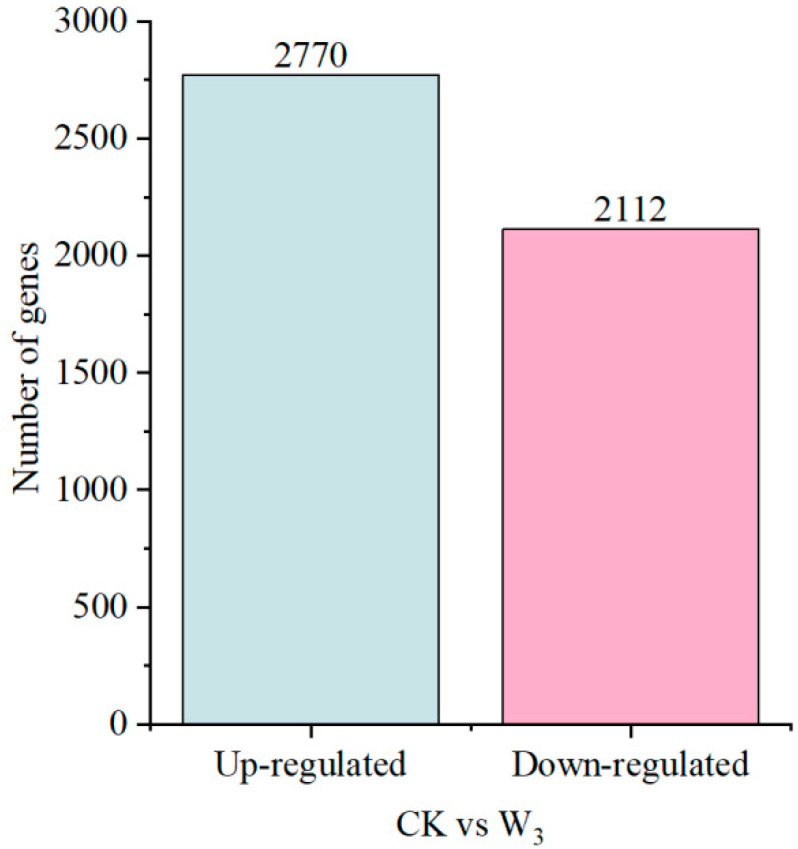
Number of differentially expressed genes (DEGs).

To explore changes in gene abundance and expression profiles under drought stress, the clean reads of RNA-Seq were aligned to the reference transcriptome. Under severe drought stress, 4882 differentially expressed transcripts (|log2FC| ≥ 1, FDR < 0.05) were identified, with 2770 up-regulated genes and 2112 down-regulated genes (Figure 5).

**Figure 6:**
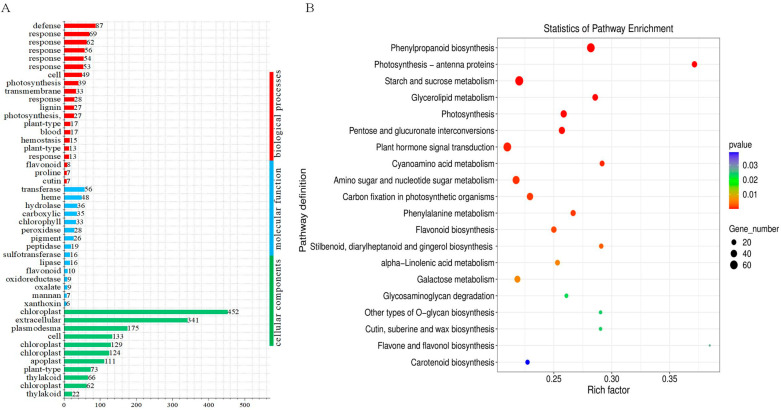
Shows the GO and KEGG analysis of DEGs of *H. halodendron* under drought stress. (**A**) GO enrichment analysis of DEGs revealed significant pathways related to drought response. (**B**) KEGG enrichment analysis of DEGs.

The GO enrichment analysis of DEGs revealed enrichment in several pathways related to photosynthesis. Specifically, 452 DEGs were enriched in the “photosynthesis, chlorophyll a/b-light harvesting” pathway, 129 DEGs in the “chloroplast thylakoid membrane” pathway, and 27 DEGs in the “chloroplast” pathway, including “photosynthesis, light reaction I” (Figure 6A). Additionally, pathways related to osmoregulation and oxidative stress showed enrichment, such as the “proline biosynthesis process” “response to fructose” “response to sucrose” “peroxidase activity” and “hydroperoxide metabolism” pathways. This suggests that the synthesis of osmotic regulatory substances and the clearance of active oxygen species play a role in helping *H. halodendron* resist drought stress (Figure 6A).

To further reveal the functional differences between these two groups of DEGs, we conducted a KEGG metabolic pathway enrichment analysis. Pathways such as “photosynthesis-photosystem I” “photosynthesis” “carbon fixation in photosynthetic organisms” “starch and sucrose metabolism” “galactose metabolism” “flavonoid biosynthesis” were significantly enriched, indicating that they are very effective in responding to drought stress (Figure 6B).

### 3.10. Identification of DEGs in Response to Photosynthesis

**Figure 7:**
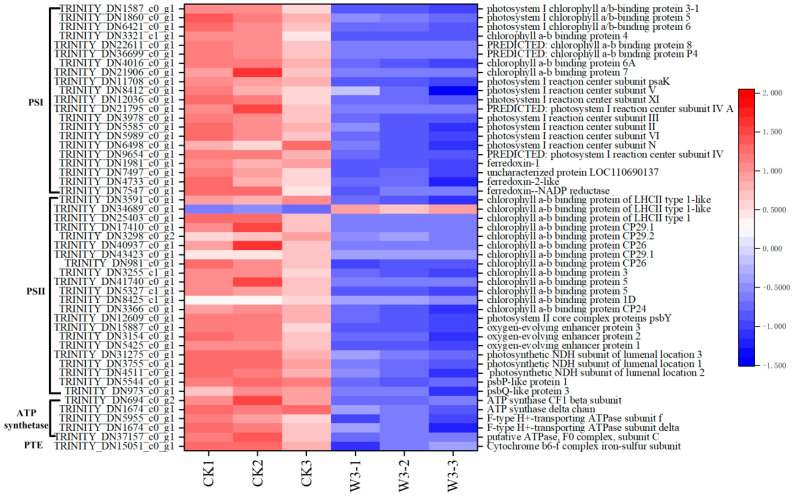
Shows studies on genes related to light-responsive of *H. halodendron* under drought stress.

**Figure 8:**
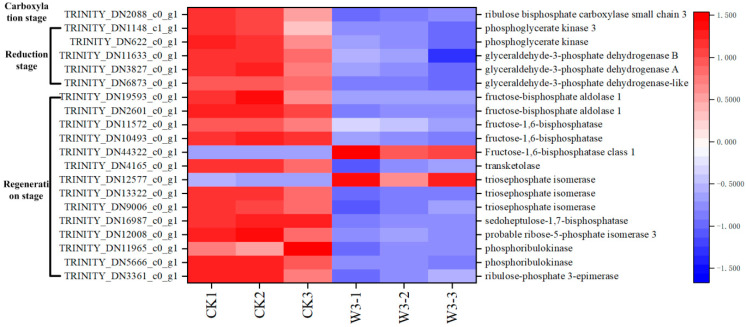
Shows studies on genes related to carbon reaction-related of *H. halodendron* under drought stress.

**Figure 9:**
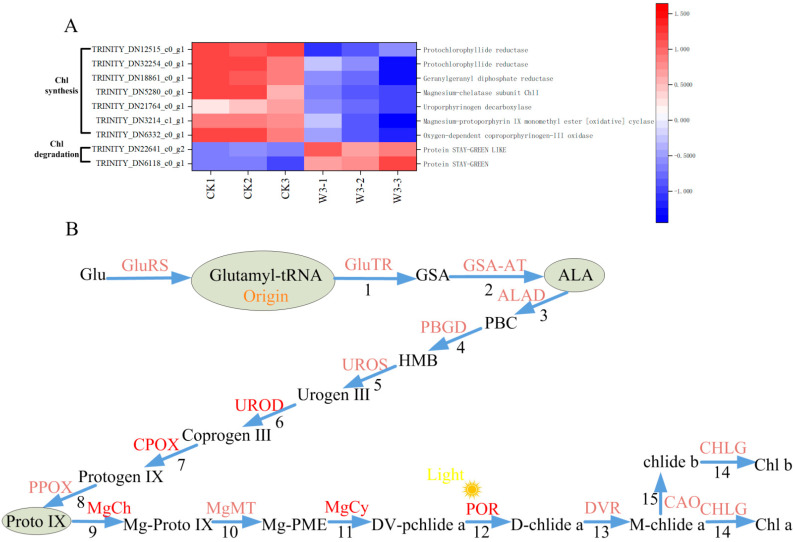
Shows studies on genes related to chlorophyll synthesis and degradation of *H. halodendron* under drought stress. (**A**) Analysis of genes involved in chlorophyll synthesis and degradation. (**B**) chlorophyll synthesis process.

Under drought stress, 49 genes involved in photosynthesis were identified as differentially DEGs. These genes included 21 involved in Photosystem I, comprising 8 chlorophyll a-b binding proteins (*Lhca*s), 9 reaction center complexes (*PSA*s), and 4 ferredoxin-oxidoredutases (*FNR*s); 22 involved in Photosystem II, comprising 13 chlorophyll a-b binding proteins (*Lhcb*s), 1 reaction center complex (*PSB*), and 8 cytochrome b6-f complex proteins; 5 ATP synthase complexes; and 1 cytochrome b6-f complex (*PET*) (Figure 7). In addition, 20 DEGs were identified involved in the carbon reaction of photosynthesis, including ribulose-1, 5-bisphosphate carboxylase/oxygenase (Rubisco), 5 enzymes involved in the reduction of glyceraldehyde-3-phosphate, and 14 enzymes regulating the regeneration of 3-phosphoenolpyruvate (Figure 8). These enzymes maintain carbon fixation and the production of organic matter in photosynthetic organisms under drought stress. Finally, we identified 7 genes that regulate chlorophyll synthesis and 2 genes that regulate its degradation as DEGs (Figure 9). The figures clearly show that the expression levels of most genes involved in photosynthesis are downregulated, aligning with physiological indicators associated with photosynthesis.

### 3.11. Identification of DEGs Related to Osmotic Regulation

**Figure 10:**
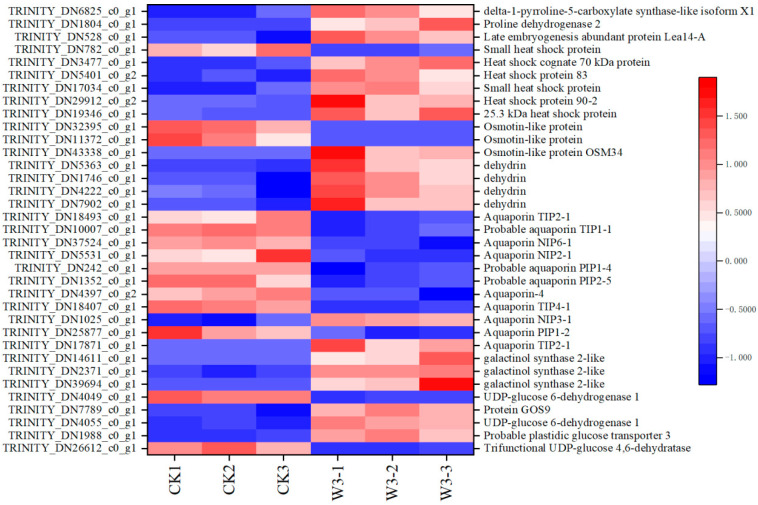
Shows studies on genes related to osmoregulation of *H. halodendron* under drought stress.

Under drought stress, multiple genes are involved in the synthesis of osmolytes for osmotic regulation, including one DEG involved in proline synthesis and one DEG regulating its degradation. Additionally, there are 8 DEGs involved in the synthesis of SS as an osmolyte to cope with drought stress. A total of 25 DEGs were identified to be related to SP, including 1 embryonic leukemia antigen-related protein (*LEA*), 6 heat shock proteins (*HSP*s), 3 osmotic stress-responsive transporter proteins (*OSM*s), 4 dehydration-responsive element-binding proteins (*DHN*s), and 11 aquaporins (*AQP*s) (Figure 10).

### 3.12. Identification of DEGs Associated with Antioxidant Capacity

**Figure 11:**
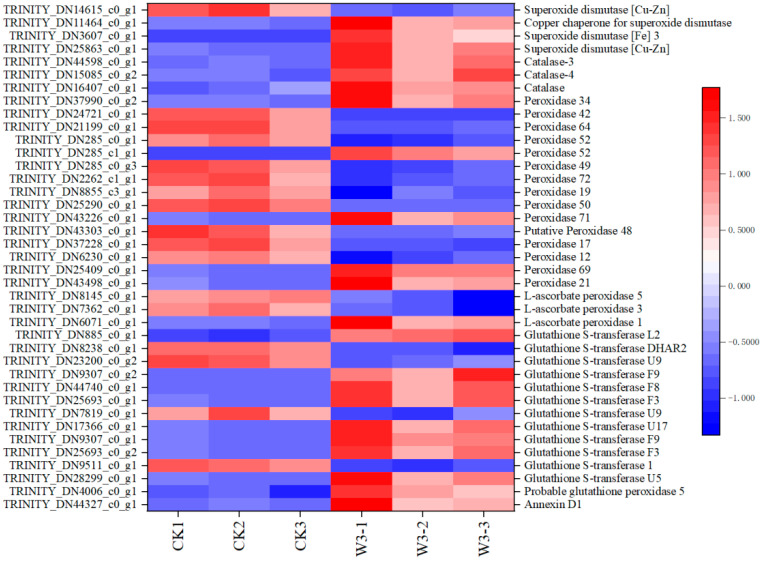
Shows studies on genes related to antioxidants of *H. halodendron* under drought stress.

A total of 39 antioxidant-related genes were identified, including 4 *SOD*s, 3 *CAT*s, 15 *POD*s, 3 ascorbate peroxidase (*APX*s), 12 glutathione-S-transferases (*GST*s), 1 glutathione peroxidase (*GPX*) and 1 attached protein (*ANN*). Moreover, the expression levels of most of these genes were up-regulated (Figure 11).

## 4. Discussion

Drought stress exerts a detrimental impact on arable land and crop production, posing a significant threat to plant growth and development. The survival of plants under drought stress is influenced not only by their structural characteristics but also by their drought tolerance and adaptability to the growing environment [14]. Studies have shown that the leaves of *H. halodendron* exhibit high water absorption rates and water accumulation, which help repair xylem embolism and enhance water conductivity, thereby conferring strong drought resistance [6]. This paper investigated the effects of varying degrees of drought stress on the drought tolerance characteristics of *H. halodendron*. It was found that *H. halodendron* employs different mechanisms to cope with different levels of drought stress. The results are as follows:

### 4.1. Effects of Drought Stress on Growth Characteristics of H. halodendron

Studies have shown that under drought stress, plants reduce leaf area and growth rates to decrease transpiration and improve water use efficiency. Drought stress can also impair mitosis, affect cell elongation and development, leading to reduced seedling height and basal diameter [15,16,17]. Analysis of this study’s results revealed that with increasing drought severity, *H. halodendron* exhibited a gradual reduction in plant height, basal diameter, number of new branches, and new branch length (Table 2). This finding suggests that *H. halodendron* similarly responds to drought stress by reducing growth rates to enhance water use efficiency.

### 4.2. Effects of Drought Stress on Photosynthetic Characteristics of H. halodendron

Photosynthesis is the process by which green plants absorb light energy to synthesize organic compounds from CO_2_ and H_2_O, releasing O_2^−^_ in the process. The majority of living organisms rely directly or indirectly on the materials and energy provided by photosynthesis for survival. Studies have shown that drought stress initially regulates ABA-induced stomatal closure, leading to a reduction in photosynthetic and transpiration rates, which severely affects plant growth and development [17]. In this study, physiological analyses revealed that as drought severity increased, the Gs of plant leaves progressively decreased (Figure 1B), resulting in reduced CO_2_ inflow, decreased photosynthesis, and a gradual decline in net Pn and Tr (Figure 1A,C). The content of Chl b began to decrease under moderate drought stress. Microscopic examination of *H. halodendron* leaves indicated that under mild drought stress, the thickness of the leaves and palisade tissue increased. However, a reduction in thickness was observed under moderate and severe drought stress. In contrast, spongy tissue showed reduced thickness even under mild drought stress (Figure 3B–D). The palisade and spongy tissues are the primary structural components of plant leaves. The palisade tissue is arranged perpendicularly to the upper epidermis cells, tightly packed, and contains numerous chloroplasts, facilitating light absorption [18]. The spongy tissue, located near the lower epidermis, has an irregular shape and loose arrangement. It possesses large intercellular spaces that form larger sub-stomatal chambers, primarily participating in gas exchange and transpiration [19]. Both tissues play crucial roles in photosynthesis. These findings suggest that under mild drought stress, the light reactions in the palisade tissue and the absorption, conversion, and transfer of light energy via Chl a and Chl b are unaffected. However, in carbon reactions, the reduced Gs and spongy tissue thickness limit CO_2_ entry into the plant, decreasing carbon fixation capacity. This decline in photosynthetic rate is primarily attributed to decreased carbon fixation and reduced synthesis of organic compounds during the carbon reaction stage. Under moderate drought stress, chloroplasts became distorted, and the thickness of the palisade tissue decreased (Figure 3I,J). The content of Chl b began to decline, affecting light absorption and electron transport, thus inhibiting the synthesis of reducing agents like NADPH and energy molecules such as ATP. This subsequently affects C3 carbon reduction and organic compound synthesis during the carbon reactions. Additionally, as the spongy tissue continued to thin and the Gs decreased further, less CO_2_ entered the plant, severely reducing carbon fixation capacity and impacting C3 reduction and organic synthesis, leading to a substantial decline in photosynthetic rate under moderate drought stress. In severe drought stress conditions, leaf mesophyll cell structures were severely damaged. Chloroplast deformation resulted in diverse shapes, and the thickness of both palisade and spongy tissues became extremely thin (Figure 3K,L). The contents of Chl a and Chl b significantly decreased, and Gs notably reduced compared to the control group, impairing light energy absorption, conversion, transfer, and organic synthesis, resulting in a marked decline in Pn.

To further elucidate the drought tolerance mechanisms, a transcriptomic analysis was conducted on *H. halodendron* leaves under both control and severe drought stress conditions. In the differential gene enrichment analyses (GO and KEGG), several pathways related to photosynthesis were enriched, and multiple genes involved in different stages of photosynthesis exhibited a downregulated expression trend, consistent with physiological data (Figure 6A,B). These results indicate that changes in photosynthesis are a crucial pathway for the drought tolerance of *H. halodendron*.

The light reactions of photosynthesis occur within the thylakoid membranes of chloroplasts [20]. Four large macromolecular subunit complexes are associated with the thylakoid membranes: Photosystem I (PSI), Photosystem II (PSII), the ATP synthase complex, and the PETs. These complexes are involved in processes such as the absorption, transfer, and conversion of light energy, electron transport, proton (H^+^) transfer, and photophosphorylation [21]. PSII functions by using the energy absorbed from light to split water molecules and transfer the released electrons to plastoquinone. It also establishes an H^+^ proton gradient across the thylakoid membrane through water oxidation and the reduction of PQB^2−^ [22]. PSI transfers electrons from plastocyanin (PC) through a series of electron acceptors to PETs and, with the help of NADP reductase, reduces NADP^+^ to NADPH, which provides energy for the dark reactions [22]. Transcriptome data identified 22 DEGs involved in PSII, including 13 *Lhcb*s, 1 *PSB*, and 8 oxygen-evolving complex proteins. Moreover, 21 DEGs were identified for PSI, including 8 *Lhca*s, 9 *PSA*s, and 4 *FNR*s (Figure 7). Among all DEGs, only the expression of Chlorophyll a-b binding protein of LHCII type 1-like (*CAB91R*, TRINITY_DN34689_c0_g1) was upregulated, while the others were downregulated. These results indicate that severe drought stress exerts substantial negative impacts on both PSI and PSII.

The functionality of PSI and PSII depends on the absorption of light energy, primarily facilitated by Chl a and Chl b, which are the key pigment molecules for light absorption and energy transfer. A small portion of Chl a is also involved in converting light energy into electrical energy [23]. Therefore, changes in Chl content significantly affect the normal progression of photosynthesis. Chl synthesis begins with the conversion of glutamyl-trna to 5-aminolevulinic acid (ALA), which is then converted into protoporphyrin IX (Proto IX) and ultimately synthesized into Chl a from Proto IX [24]. In this study, transcriptome data revealed 7 differentially expressed genes (DEGs) involved in chlorophyll synthesis (Figure 9A). Among these, *HEME* encodes UROD, and *HEMF* encodes CPOX, both of which play crucial roles in the conversion of ALA to Proto IX (Figure 9B). In the synthesis of Chl a from Proto IX, five DEGs are involved (Figure 9A), including *ChlI*, which encodes MgCh; *CRD1*, which encodes MgCy; *POR*, which catalyzes the conversion of PChlide to Chlide; and *ChlP*, which catalyzes the reduction of geranylgeranyl diphosphate to phytol diphosphate, providing phytol for chlorophyll synthesis (Figure 9B). Additionally, DEGs involved in chlorophyll degradation, *SGRL* and *SGR*, were identified (Figure 9A,B); these proteins promote chlorophyll degradation by facilitating interactions between LHCII and various chl-synthesizing enzymes. Gene expression analysis showed that the expression levels of all genes involved in chlorophyll synthesis were downregulated, while those involved in degradation were upregulated (Figure 9A). These findings indicate that drought stress impedes chl synthesis in the leaves of *H. halodendron* and accelerates its degradation, leading to reduced chlorophyll content. Consequently, this affects the absorption, transfer, and conversion of light energy, which aligns with physiological indicators.

The *PET*s primarily facilitate the transfer of electrons from PSII to PSI and catalyze the translocation of H^+^ ions across the membrane, creating a transmembrane electrochemical proton gradient. This gradient provides the energy necessary for the synthesis of adenosine triphosphate (ATP) [25]. ATP synthase catalyzes the formation of ATP from the combination of inorganic phosphate (Pi) and adenosine diphosphate (ADP) using the energy released when H^+^ ions move back across the membrane along the electrochemical gradient, supplying energy for the dark reactions [26]. In this study, five ATP synthase-related genes involved in ATP synthesis and one *PET*s-related gene responsible for electron transfer were identified (Figure 7), all of which showed a trend of downregulated expression. These results suggest that severe drought stress causes a reduction in chlorophyll synthesis and accelerates its degradation in the leaves of *H. halodendron*, leading to decreased chlorophyll content and reduced activity of LHC proteins. Consequently, the absorption, transfer, and conversion of light energy are impaired, affecting electron transfer and the synthesis of NADPH and ATP. NADPH and ATP provide the energy essential for carbon reactions; their inhibited synthesis further impacts the reduction of C3 and the synthesis of organic compounds in these reactions.

The carbon reactions in photosynthesis are divided into three stages: carboxylation, reduction, and regeneration. Under drought stress, the earliest response is the closure of stomata induced by ABA, which reduces the influx of CO_2_ and subsequently limits carboxylation, thereby affecting both the reduction and regeneration stages [27]. In the C3 pathway, the acceptor of CO_2_ during the carboxylation stage is ribulose-1,5-bisphosphate (RuBP), and the enzyme catalyzing this reaction is ribulose-1,5-bisphosphate carboxylase/oxygenase (Rubisco, EC:4.1.1.39) [20]. In this study, one Rubisco gene involved in CO_2_ fixation was identified, but it showed a trend of downregulated expression (Figure 8). Due to the reduced internal CO_2_ levels, the carboxylation process is constrained, leading to a decrease in carbon fixation and an obstruction in the synthesis of the initial product, glycerate-3-phosphate (Glycerate-3P), during the reduction stage.

Additionally, we identified five differentially expressed genes (DEGs) involved in the reduction of Glycerate-3P to glyceraldehyde-3-phosphate (Glyceraldehyde-3P), all of which exhibited downregulated expression (Figure 8). This finding suggests that the reduction of Glyceraldehyde-3P is impeded, leading to reduced levels of starch and sucrose. Consequently, this impacts plant growth and development, resulting in slower growth. Under severe drought stress conditions, the study observed reduced plant height, slow new shoot growth, and decreased plant dry weight, which are consistent with these findings. Glyceraldehyde-3P is regenerated into RuBP via a series of enzymatic reactions, continuing CO_2_ fixation. However, the hindrance in the reduction of Glyceraldehyde-3P, along with the downregulation of most of the 14 identified genes encoding RuBP regeneration enzymes (Figure 8), suggests that RuBP regeneration is also inhibited.

The reduction and regeneration stages require the energy provided by NADPH and ATP produced during the light reactions. However, their synthesis is also obstructed under drought stress, affecting energy availability. Therefore, severe drought stress disrupts the Calvin cycle, negatively impacting CO_2_ fixation, the reduction of Glyceraldehyde-3P, and the regeneration of RuBP. This results in impaired synthesis and reduced accumulation of organic compounds, with significant adverse effects on the growth and development of *H. halodendron*.

### 4.3. The Regulation of Osmosis Related to H. halodendron Gene Expression

Under drought stress, stomatal closure reduces Tr and increases WUE, thereby preventing osmotic stress. This study observed that as drought stress intensified, the Gs of *H. halodendron* continuously decreased, Tr decreased, and WUE increased (Figure 1B–D). Research indicates that *H. halodendron* possesses a high leaf water uptake rate and water accumulation capability, contributing to higher WUE [6]. These results suggest that *H. halodendron* can mitigate drought damage by reducing water evaporation and enhancing water uptake capacity to adjust to osmotic stress.

One direct response of plants to drought stress is the accumulation of osmotic adjustment substances, such as free amino acids and SS, to cope with osmotic stress. Studies have shown that drought stress can increase Pro content by up to 100 times the normal level, composing 80% of the total amino acid pool in many plants, and the accumulation of Pro helps maintain relative water content in plants [1]. Moreover, Pro can alleviate oxidative stress by scavenging ROS, thus reducing membrane damage [1]. In this study, Pro was found to significantly accumulate under severe drought stress (Figure 2B), and the expression of the gene *P5CS*, which regulates Pro synthesis, was upregulated (Figure 10), accelerating Pro synthesis and increasing its content. As Pro breakdown slows, Pro content increases. Additionally, the expression of the gene *POX2*, which regulates Pro degradation, was found to trend upwards, indicating accelerated Pro breakdown. Research suggests that *POX2* can generate ROS during Pro degradation, speeding up cellular senescence [28]. Based on these findings, it is hypothesized that under severe drought stress, the rate of Pro synthesis exceeds its degradation rate. The accumulation of a substantial amount of Pro not only alleviates osmotic stress but also helps eliminate the ROS generated during Pro degradation, thereby reducing cellular damage.

Drought stress induces both qualitative and quantitative changes in plant proteins. In this study, the SP content significantly increased under all levels of drought stress, with a dramatic rise under severe drought conditions (Figure 2D). Transcriptomic analysis identified several SPs involved in osmotic regulation, including *LEA*s, *HSP*s, *OSM*s, *DHN*s, and *AQP*s (Figure 10). Research has indicated that *LEA*s, *HSP*s, *OSM*s, and *DHN*s can protect cells from drought stress [29]. *LEA*s are hydrophilic proteins that protect other proteins under drought stress conditions [30]. *OSM*s help safeguard cells from high osmotic pressure and structural or metabolic disruption [31]. Multiple *HSP*s have been shown to be beneficial in resisting drought stress; some genes enhance drought resistance by boosting antioxidative enzyme activity, reducing ROS, and upregulating stress-related genes [32,33,34,35]. *DHN*s enhance drought stress tolerance by increasing water retention, maintaining chlorophyll content, preserving the photosynthetic machinery, activating ROS detoxification, and promoting the accumulation of compatible solutes [36]. The observed upregulation trend in the expression of genes encoding these four proteins suggests that they protect *H. halodendron* cells from drought stress through various mechanisms.

*AQPs* play a crucial role in plant water balance and effective water use [37,38,39]. Under drought conditions, *AQPs* can affect crop physiological performance by influencing water transport and, consequently, Gs [40]. In this study, Gs and Tr continually decreased under drought stress, affecting water flow controlled by *AQPs*. Transcriptomic analysis identified 11 *AQPs* involved in intracellular and extracellular water flow, with 9 of them showing downregulated expression (Figure 10). These findings suggest that *H. halodendron* may reduce *AQP* activity under drought stress to influence Gs and Tr, thereby lowering water utilization rates. This adjustment allows for sustained physiological activities despite the stress. By modulating *AQP* expression, the plant can optimize water use efficiency and maintain essential physiological processes during drought conditions.

SSs function as osmotic regulators by reducing water loss in plants and increasing cell turgor to cope with osmotic stress. Sugars have also been found to activate antioxidant processes, emerging as novel ROS scavengers [41]. Trehalose is known to protect photosystem II from oxidative stress and regulate ABA during plant stress [41]. Increases in raffinose concentration have been shown to enhance the ability of transgenic *Arabidopsis* to scavenge ROS [1]. In this study, the content of soluble sugars significantly increased under moderate and severe drought stress, aiding in osmotic regulation (Figure 2C). Transcriptomic data revealed the upregulation of genes involved in the synthesis of raffinose and trehalose, leading to higher soluble sugar levels, consistent with physiological indicators (Figure 10). These results suggest that *H. halodendron* can alleviate drought damage by increasing SS content to regulate osmotic stress and enhance antioxidant capacity, thereby protecting the plant from the adverse effects of drought.

### 4.4. Expression of Antioxidation Genes in H. halodendron

Under drought stress, the decline in stomatal conductance and CO_2_ uptake leads to an over-reduction of the electron transport system and carbon starvation in organelles, resulting in excessive ROS production that induces oxidative damage to various important macromolecules, thus limiting plant growth and development [42]. This study measured the production rate of O_2^−^_ under varying levels of drought stress, revealing that as drought severity increases, the O_2^−^_ production rate accelerates, especially under severe drought stress (Figure 2F). This significantly impacts internal structures in *H. halodendron*, such as DNA, proteins, and cell membranes. Elevated ROS and peroxide levels can lead to lipid peroxidation, reduced cell integrity, electrolyte leakage, and cellular toxicity [43]. As drought severity increased, the relative electrolyte leakage also intensified, indicating progressively compromised cell membrane integrity. Research has shown that virtually all subcellular organelles possess antioxidant systems to ameliorate and clear ROS, thus mitigating oxidative stress [42]. In this study, transcriptomic data identified several antioxidant enzymes involved in regulating oxidative stress, including 4 *SOD*s, 3 *CAT*s, 15 *POD*s, 3 *APX*s, 12 *GST*s, 1 *GPX*, and 1 *ANN* (Figure 11). Additionally, the activities of SOD, CAT, and POD were measured (Figure 2G–I). SOD and CAT activities were significantly elevated under all levels of drought stress, while POD activity increased significantly only under severe drought stress. This indicates that SOD and CAT maintain high activity to regulate oxidative stress and scavenge reactive oxygen species, whereas POD primarily functions under severe drought stress. Although the activities of all three antioxidant enzymes increased significantly under severe drought stress, the rapid production of ROS overwhelmed the antioxidant balance. This led to severe damage to cell membrane integrity, increased electrolyte leakage, and, consequently, severely affected plant growth and development.

## 5. Conclusions

In summary, this study conducted a comprehensive analysis of the physiological, biochemical, and transcriptomic responses of *H. halodendron* under drought stress. The results showed that drought stress significantly reduced the net photosynthetic rate, stomatal conductance, transpiration rate, and chlorophyll content while increasing the content of Pro, SS, and SP and enhancing the activity of antioxidant enzymes such as SOD, CAT, and POD. Through transcriptomic data analysis, we identified 49 DEGs involved in light reactions, 20 DEGs involved in carbon reactions, and 11 DEGs related to chlorophyll synthesis and degradation. Additionally, we identified 35 DEGs associated with osmotic regulation, including 2 related to proline synthesis and degradation, 8 involved in SS synthesis, and 25 related to SP.

In terms of antioxidation, 39 related DEGs were identified, comprising 4 SODs, 3 CATs, 15 PODs, 3 APXs, 12 GSTs, 1 GPX, and 1 ANN. These findings provide important insights into the physiological and molecular response mechanisms of *H. halodendron* under drought stress.

## Figures and Tables

**Table 1:** Soil moisture gradient setting.

Water Treatment	Field Capacity/%	Soil Mass Water Content/%
CK (control treatment)	80~90	16.25~18.28
W_1_ (Mild drought)	60~70	12.19~14.22
W_2_ (Moderate drought)	40~50	8.12~10.16
W_3_ (Severe drought)	20~30	4.06~6.09

**Table 2:** Effects of drought stress on growth index of *H. halodendron*.

Stress Time	Stress Gradient	Increment of Plant Height/cm	Increment of Ground Diameter/mm	The Number of New Shoots Increases/Branch	Increment of New Branch Length/cm
30d	CK	9.87 ± 2.29 a	2.08 ± 0.56 a	3.00 ± 1.00 a	7.6 ± 1.04 a
	W_1_	6.17 ± 1.56 b	2.09 ± 0.82 a	3.00 ± 1.73 a	6.13 ± 0.50 b
	W_2_	4.10 ± 1.06 bc	1.20 ± 0.38 ab	2.00 ± 0.00 a	5.1 ± 0.72 b
	W_3_	2.47 ± 1.27 c	0.49 ± 0.35 b	1.33 ± 0.58 a	2.27 ± 0.49 c

Note: Analysis of variance (ANOVA) was used as the statistical method. Different lowercase letters indicate significant differences between treatments (*p* < 0.05).

**Table 3:** Effects of drought stress on the dry weight of each organ of *H. halodendron*.

Stress Time	Stress Gradient	Dry Weight of Root/g	Dry Weight of Root Stem/g	Dry Weight of Leaf/g	Dry Weight of Whole Plant/g	Root-Shoot Ratio
30d	CK	1.57 ± 0.36 a	1.71 ± 0.27 ab	2.12 ± 0.44 a	5.39 ± 0.98 a	0.41 ± 0.08 a
	W_1_	1.25 ± 0.21 ab	2.05 ± 0.06 a	2.27 ± 0.58 a	5.58 ± 0.81 a	0.29 ± 0.03 a
	W_2_	1.14 ± 0.24 ab	1.34 ± 0.27 bc	1.88 ± 0.33 a	4.36 ± 0.65 ab	0.36 ± 0.09 a
	W_3_	0.96 ± 0.36 b	0.98 ± 0.09 c	1.08 ± 0.29 b	3.01 ± 0.57 b	0.46 ± 0.13 a

Note: Analysis of variance (ANOVA) was used as the statistical method. Different lowercase letters indicate significant differences between treatments (*p* < 0.05).

**Table 4:** Effects of drought stress on the microstructure of *H. halodendron*.

Stress Time	Stress Time	Blade Thickness/μm	Palisade Tissue Thickness/μm	Spongy Tissue Thickness/μm	Main Vascular Bundle Thickness/μm
30d	CK	747.57 ± 84.47 a	337.54 ± 14.39 a	302.09 ± 27.93 a	335.27 ± 40.00 b
	W_1_	804.26 ± 37.27 a	352.70 ± 14.29 a	230.65 ± 14.05 b	481.14 ± 114.55 a
	W_2_	488.18 ± 7.60 b	211.96 ± 8.66 b	146.06 ± 15.82 c	302.94 ± 19.55 b
	W_3_	411.74 ± 59.81 b	162.87 ± 43.40 c	99.29 ± 7.02 d	292.18 ± 33.13 b

**Table 5:** Summary of Illumina transcripts.

	Transcript	Unigenes
Min Length	179 bp	201 bp
Max Length	15,719 bp	15,719 bp
Median Length	1017 bp	505 bp
N50	1888 bp	1632 bp
GC%	40.29	41.23
Q30	91%	91%

## Data Availability

The accession number of the transcriptome data in NCBI is SUB15419086.

## Abbreviations

abbreviations	Full name
DEGs	differentially expressed genes
SOD	superoxide dismutase
O_2^−^_	superoxide anion
CAT	catalase
POD	peroxidase
Pn	net photosynthetic rate
Tr	transpiration rate
Gs	stomatal conductance
WUE	water use efficiency
Chl a	Chlorophyll a
Chl b	chlorophyll b
PSI	Photosystem I
PSII	Photosystem II
RWC	relative water content
REC	conductivity
OFR	Oxygen free radical
Pro	proline
SS	soluble sugar
SP	soluble protein
*Lhca*s	chlorophyll a-b binding proteins
*PSA*s	reaction center complexes
*FNR*s	ferredoxin-oxidoredutases
*Lhcb*s	chlorophyll a-b binding proteins
*PSB*	reaction center complex
*PET*	cytochrome b6-f complex
Rubisco	ribulose-1, 5-bisphosphate carboxylase/oxygenase
RuBP	ribulose-1,5-bisphosphate
*LEA*	embryonic leukemia antigen-related protein
*HSP*s	heat shock proteins
*OSM*s	osmotic stress-responsive transporter proteins
*DHN*s	dehydration-responsive element-binding proteins
*AQP*s	aquaporins
*APX*s	ascorbate peroxidase
*GST*s	glutathione-S-transferases
*GPX*	glutathione peroxidase
*ANN*	attached protein
PC	plastocyanin
ALA	5-aminolevulinic acid
Proto IX	protoporphyrin IX
ATP	adenosine triphosphate
ADP	adenosine diphosphate
Pi	inorganic phosphate
Glycerate-3P	glycerate-3-phosphate
Glyceraldehyde-3P	glyceraldehyde-3-phosphate
